# Supplementary figures and images for: Ultrasound-assisted magnetic nanoparticle-based gene delivery
Source: PLoS One. 2020 Sep 24;15(9):e0239633. doi: 10.1371/journal.pone.0239633 (PMC7514102; doi:10.1371/journal.pone.0239633)

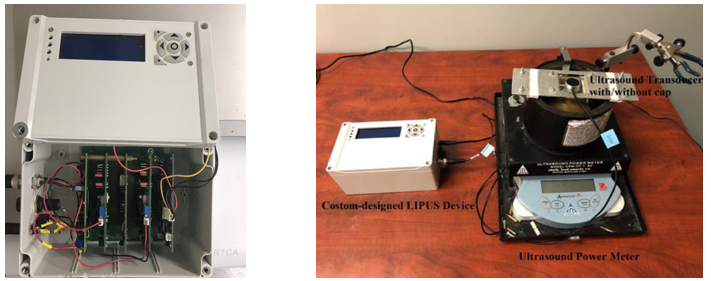


(a) (b)

S1 Fig: (a) LIPUS generation box, (b) photograph of the setup for measuring acoustic impedance.

Supplement: S1 Fig — (DOCX) [file pone.0239633.s001.docx]

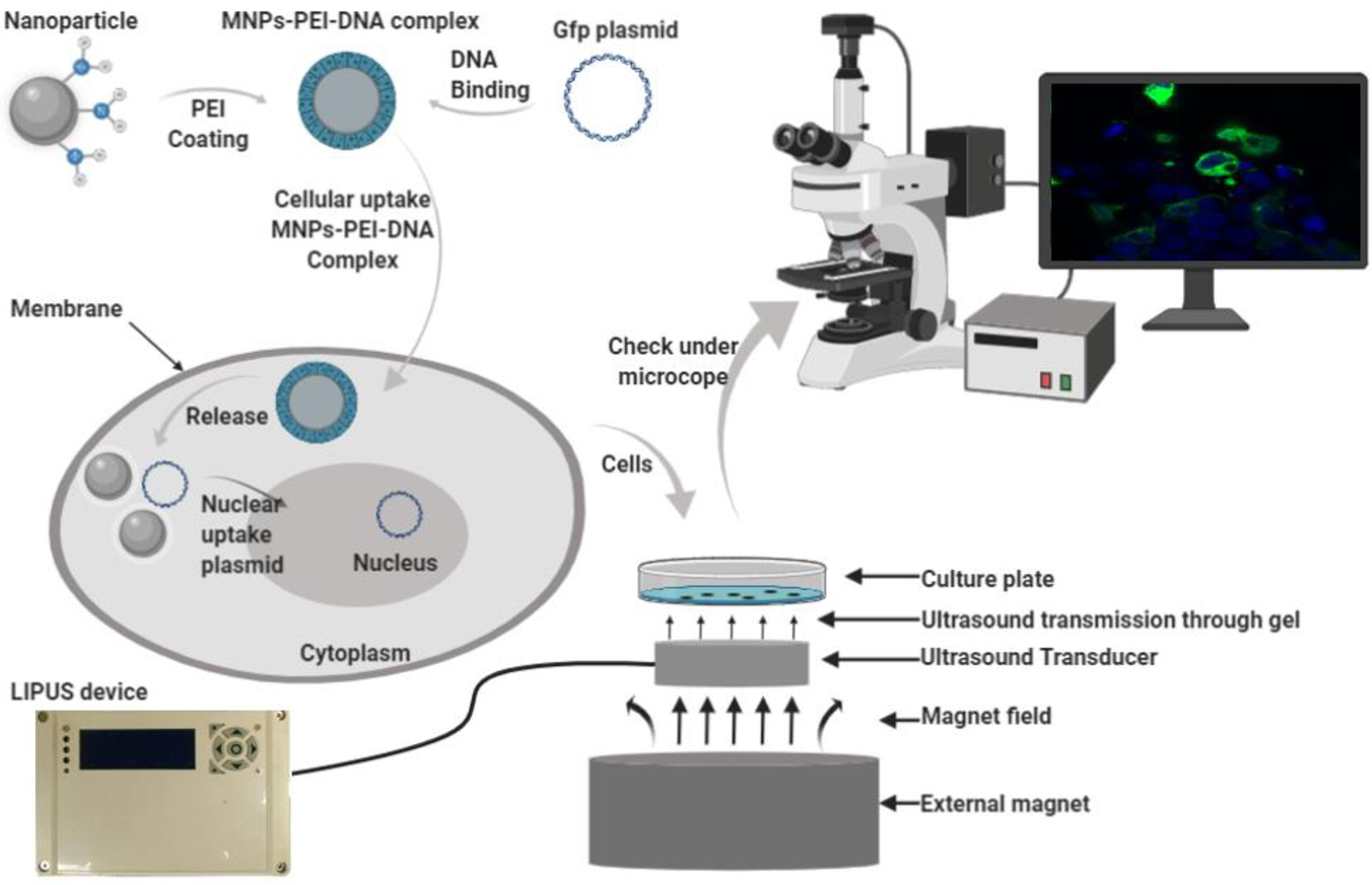

Supplement: S1 Graphical abstract — (TIF) [file pone.0239633.s007.tif]
