# Supplementary material for: Ultrasound-assisted magnetic nanoparticle-based gene delivery
Source: PLoS One. 2020 Sep 24;15(9):e0239633. doi: 10.1371/journal.pone.0239633 (PMC7514102; doi:10.1371/journal.pone.0239633)
Supplement: S3 Fig — (a) lipofectamine 2000, (b) our MNPs and magnet, (c) our suggested method: MNPs, magnet, in combination with LIPUS treatment. (DOCX) [file pone.0239633.s003.docx]

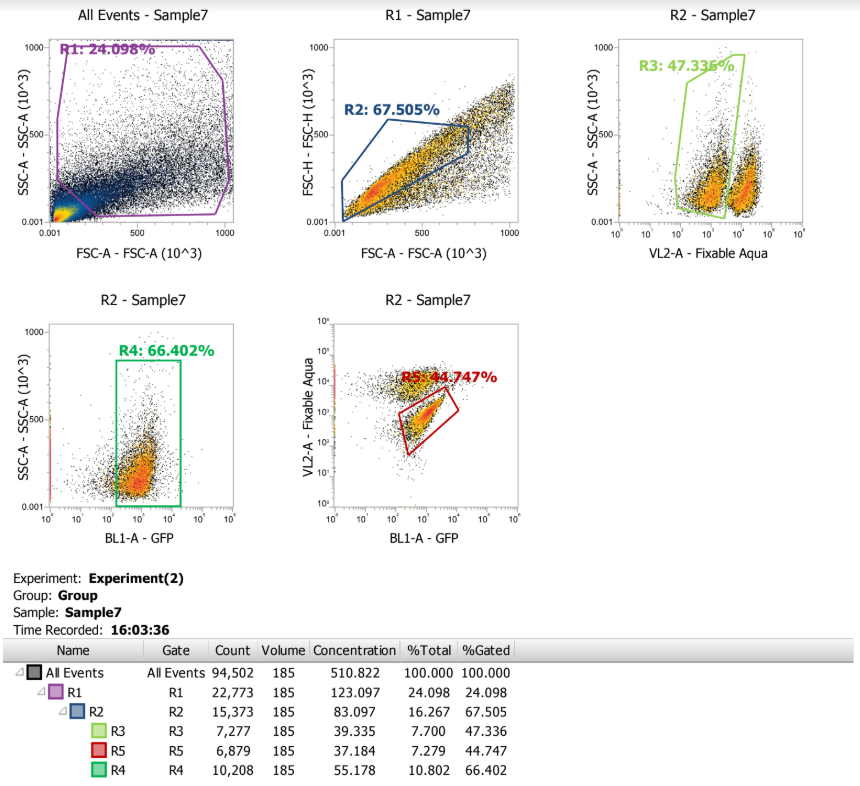


(a)


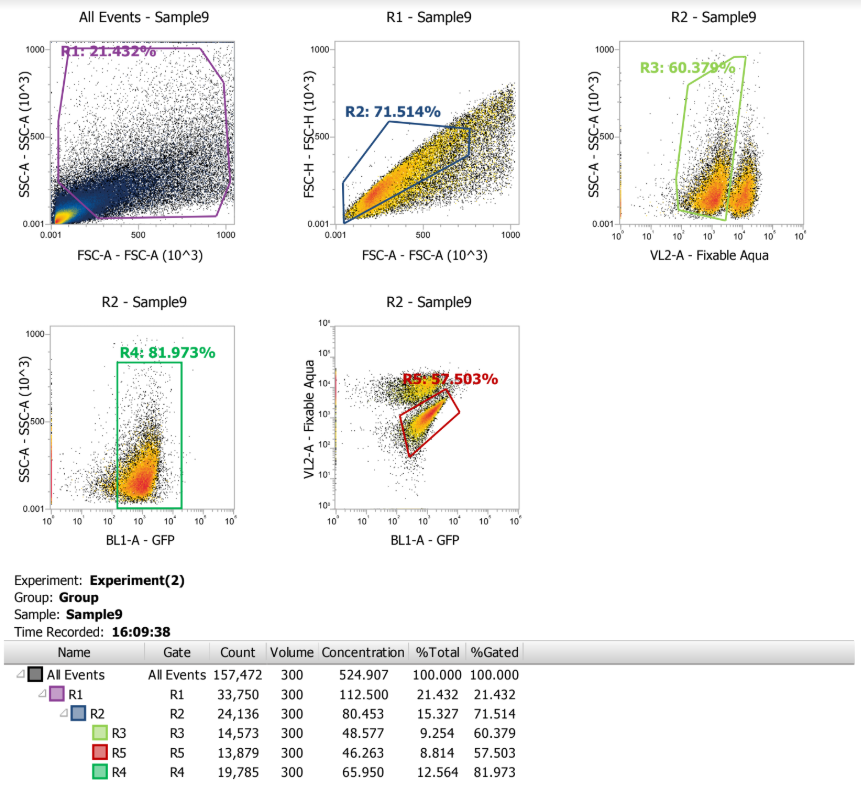


(b)


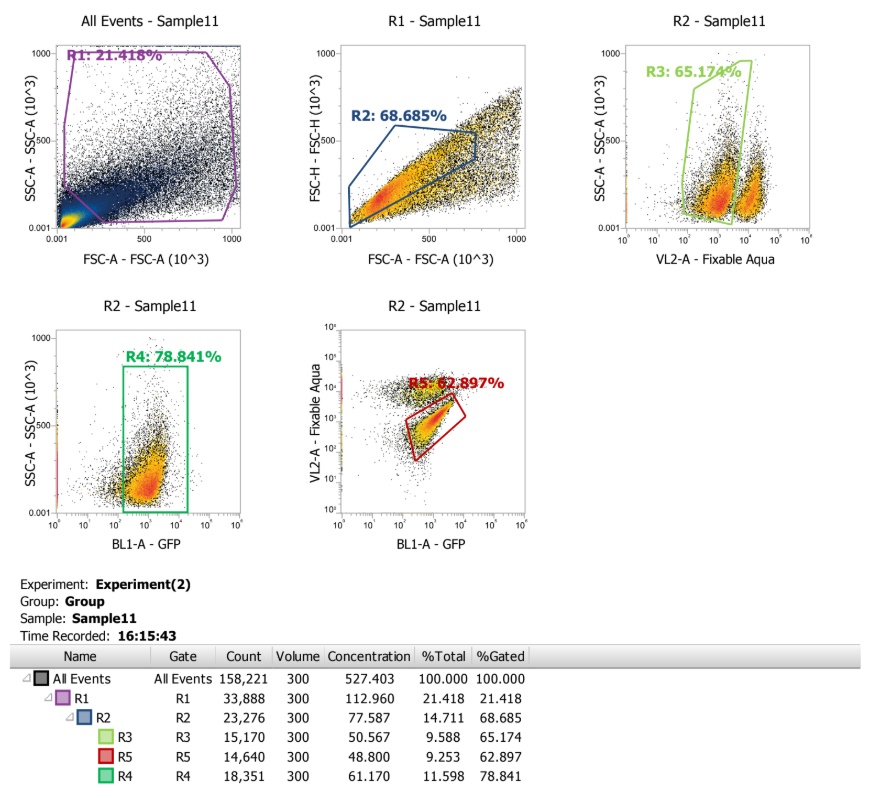


(c)

S3 Fig: Another example of quantification of transfection: (a)-(c) show the flow cytometry histogram plots of transfection rates and cell viability using GFP with different methods. (a) lipofectamine 2000, (b) our MNPs and magnet, (c) our suggested method: MNPs, magnet, in combination with LIPUS treatment.
