# Supplementary material for: Ultrasound-assisted magnetic nanoparticle-based gene delivery
Source: PLoS One. 2020 Sep 24;15(9):e0239633. doi: 10.1371/journal.pone.0239633 (PMC7514102; doi:10.1371/journal.pone.0239633)
Supplement: S4 Fig — (DOCX) [file pone.0239633.s004.docx]

S4 Fig: Cell proliferation after stimulation with LIPUS

under 10 different intensity and duration parameters.
