# Supplementary material for: Ultrasound-assisted magnetic nanoparticle-based gene delivery
Source: PLoS One. 2020 Sep 24;15(9):e0239633. doi: 10.1371/journal.pone.0239633 (PMC7514102; doi:10.1371/journal.pone.0239633)
Supplement: S1 Table — (DOCX) [file pone.0239633.s005.docx]

S1 Table: Cell proliferation after stimulation with LIPUS

under 10 different intensity and duration parameters.

|  | 5 mins | | | | 10 mins | | | |
| --- | --- | --- | --- | --- | --- | --- | --- | --- |
| 0mW/cm2 | 800000/mL | | 900000/mL | | 820000/mL | | 780000/mL | |
| 10mW/cm2 | 540000 | 420000 | 400000 | 280000 | 440000 | 340000 | 320000 | 200000 |
| 20mW/cm2 | 380000 | 440000 | 340000 | 380000 | 500000 | 480000 | 660000 | 500000 |
| 30mW/cm2 | 400000 | 340000 | 300000 | 240000 | 1040000 | 860000 | 860000 | 720000 |
| 40mW/cm2 | 800000 | 1000000 | 980000 | 820000 | 540000 | 680000 | 640000 | 480000 |
| 60mW/cm2 | 380000 | 400000 | 320000 | 340000 | 460000 | 720000 | 460000 | 440000 |
