# Supplementary material for: Ultrasound-assisted magnetic nanoparticle-based gene delivery
Source: PLoS One. 2020 Sep 24;15(9):e0239633. doi: 10.1371/journal.pone.0239633 (PMC7514102; doi:10.1371/journal.pone.0239633)
Supplement: S2 Table — (DOCX) [file pone.0239633.s006.docx]

S2 Table: Cell proliferation after stimulation with LIPUS

under 4 different intensity and duration parameters.

|  | 5 mins | | | | 10 mins | | | |
| --- | --- | --- | --- | --- | --- | --- | --- | --- |
| 0mW/cm2 | 110000 | | 120000 | | 95000 | | 85000 | |
| 30mW/cm2 | 80000/mL | 150000/mL | 100000/mL | 80000/mL | 155000/mL | 160000/mL | 140000/mL | 180000/mL |
| 40mW/cm2 | 120000/mL | 120000/mL | 150000/mL | 125000/mL | 105000/mL | 145000/mL | 120000/mL | 90000/mL |
